# Balancing one’s own life with caregiving for a sibling with schizophrenia: A qualitative study

**DOI:** 10.1016/j.ijnsa.2026.100590

**Published:** 2026-06-04

**Authors:** Megumi Kawaguchi, Miho Katayama, Akiyo Nakamoto, Hiromi Morioka, Midori Kawamura

**Affiliations:** a Division of Nursing, Faculty of Medical Sciences, University of Fukui, Fukui, Japan; b Department of Nursing, Faculty of Health Sciences, Komatsu University, Ishikawa, Japan; c Department of Nursing, Faculty of Nursing and Rehabilitation, Konan Women's University, Kobe, Japan; d Department of Nursing, Faculty of Health Sciences, Kio University, Nara, Japan; e Department of Nursing Science, Faculty of Nursing & Social Welfare Sciences, Fukui Prefectural University, Fukui, Japan

**Keywords:** Family caregivers, Adult sibling relationships, Schizophrenia

## Abstract

**Background:**

Siblings of individuals with schizophrenia often assume caregiving roles as their parents age. However, the process by which the unaffected siblings balance their family life and their relationship with the affected sibling remains unclear.

**Objective:**

This study aimed to clarify the process by which married individuals reconstruct their relationship with a sibling who has schizophrenia as their parents age.

**Design:**

This study employed a qualitative design based on the modified grounded theory approach.

**Setting:**

The participants were recruited through mental health and family welfare associations by snowball sampling.

**Participants:**

The participants were 7 married individuals (1 man, 6 women; age, 50–65 years) who had a sibling with schizophrenia.

**Methods:**

Data were collected through semi-structured interviews with the participants, and verbatim transcripts were analyzed to generate concepts and categories.

**Results:**

The analysis revealed a dynamic process in which married individuals who have a sibling with schizophrenia transition from self-sacrificing involvement to a relationship of “running alongside” the affected sibling. Initially, the unaffected siblings **[Feel overwhelmed by a sense of duty]**. As caregiving burdens threaten their own family life, they reach a critical turning point where they **[Protect life with their partner]**. Following this decision, the unaffected siblings establish boundaries by which they **[Maintain a comfortable distance from the affected sibling]**. Concurrently, through peer interactions, they **[*Re*-examine their identity]**.

**Conclusions:**

The unaffected siblings’ decision to protect their own families is a crucial turning point where they recognize the limits of their self-sacrifice. Acknowledging that they cannot fully assume the parental role, they establish a sustainable distance and reconstruct their identities through peer support. Ultimately, they transition to “running alongside” the affected sibling. Nursing professionals should facilitate this transition by providing psychological support, connecting them with social resources, and offering opportunities for peer interactions.

**Study registration:**

Not registered.

**What is already known**

• Adult siblings of individuals with schizophrenia need to assume caregiving responsibilities for the affected sibling.
• The process through which they balance their own family life and caregiving responsibilities warrants greater attention.

Alt-text: Unlabelled box dummy alt text

**What this paper adds**

• This study found that the process involves transitioning from self-sacrifice to sustainable caregiving.
• Peer interactions help individuals develop positive self-perception and reconstruct their identity.
• Relevant information, psychological support, social resources, and peer interaction are crucial for individuals providing care to siblings with schizophrenia.

Alt-text: Unlabelled box dummy alt text

## 1 Background

Schizophrenia is a major global health concern that affects 24 million individuals worldwide (World Health Organization, 2025). Its onset typically occurs between adolescence and young adulthood (World Health Organization, 2025), typically requiring parents to assume primary caregiving roles. The relapse rate of schizophrenia is high, with symptoms worsening over time in some patients (Harrison et al., 2001) and necessitating long-term support. Studies on the parents of individuals with schizophrenia have shown that the parents often feel deprived of their rights (Milliken, 2001) and experience pressure (Maan et al., 2024) and social stigma (Manesh et al., 2023). They also experience difficulties in accepting the diagnosis (Forcheron et al., 2023). Moreover, older parents face challenges such as barriers to social and family interactions and the inability to effectively respond to the needs of an adult child with schizophrenia (Sharifi et al., 2023).

Given the typical age of onset of schizophrenia, siblings without schizophrenia often have to live with an affected sibling. Research has shown that these unaffected siblings experience shame regarding the diagnosis, lack knowledge of support resources, and face social isolation (Graves et al., 2020). As their parents age, these siblings take on caregiving roles, including medical appointment assistance, medication management, and financial support (Amaresha et al., 2019; Steel, 2019). Therefore, establishing support systems for the siblings of individuals with schizophrenia is crucial.

Research on the siblings of individuals with disabilities has predominantly focused on congenital or childhood-onset conditions such as intellectual disabilities, physical disabilities, or developmental disorders. Studies have shown that growing up with a sibling with a disability causes unaffected siblings to perceive their affected sibling as “normal” (Lee et al., 2025), and that the unaffected siblings are often involved in future support planning (Casale et al., 2021). Even when parents state that the siblings’ involvement in future care is unnecessary, the siblings take on the responsibility of providing support and implicitly prepare for future roles (Chiu, 2022). This experience influences these siblings' personality traits, religious views, career choices, and romantic relationships (Milevsky and Singer, 2022). Studies have also indicated that living with an affected sibling provides motivation in life (Kim et al., 2025).

Schizophrenia typically manifests after normal development; therefore, the experiences of individuals who have a sibling with schizophrenia may be qualitatively distinct, often involving a sense of loss for their previously healthy sibling (Gerace et al., 1993). Research on the siblings of individuals with schizophrenia has highlighted these challenges. Siblings who assume caregiving responsibilities experience anxiety regarding their current and future obligations (Yang et al., 2017). Caregiving involves significant financial, temporal, physical, and psychological pressure (Amaresha et al., 2019). Individuals caring for a sibling with schizophrenia show higher depressive symptoms than those caring for siblings with other mental disorders (Jayasinghe et al., 2023). Severe schizophrenia symptoms with minimal treatment increase sibling relationship conflicts (Plessis et al., 2020). Collectively, these findings highlight the caregiving experiences and personal distress of individuals who have a sibling with schizophrenia.

Although societal support is vital for individuals with schizophrenia, positive relationships with family members, including siblings, form a critical component of recovery (Hsiao and Tsai, 2021; Ma et al., 2023). When the siblings of individuals with schizophrenia age and marry, their priorities often shift from their original families to their new families. However, it remains unclear how married adult siblings balance these transitions and familial duties. Therefore, this study aimed to clarify the process by which married individuals reconstruct their relationship with a sibling who has schizophrenia as their parents age.

## 2 Methods

### 2.1 Research design

This study utilized Kinoshita’s (2020) modified grounded theory approach, which aims to generate theories that can be practically applied in relevant contexts within the field of human services. The modified grounded theory approach was chosen specifically because the objective of the present study was not merely to describe individuals’ static experiences of caregiver burden but to clarify the structural process underlying how married individuals reconstruct their relationship with an affected sibling while maintaining their own lives as their parents age. The modified grounded theory approach was selected for three primary reasons. First, it enables the examination of processual dynamics stemming from the various social interactions among the married individuals, their siblings with schizophrenia, caregiving parents, professionals, and support networks. Second, this approach aligns deeply with nursing, a human services field established through such social interactions. Finally, it generates highly practical theories, enabling the results to be directly implemented in clinical practice.

Furthermore, adoption of the modified grounded theory approach significantly influenced our analytical decisions. Instead of simply extracting and listing thematic categories of the participants’ experiences, this methodological framework guides researchers in focusing on the structural process based on the relationships among phenomena rather than mere chronological changes. Consequently, it drove our analytical focus toward conceptualizing how various interactions and turning points, including the shift from traditional “self-sacrifice” to a new relationship of “running alongside” the affected sibling, are structurally interconnected. This approach ultimately enabled the construction of a comprehensive process model.

### 2.2 Characteristics and reflexivity of the researcher

The primary researcher, a psychiatric nursing specialist and a mother, performed the data analysis. To ensure research transparency and rigorous reflexivity, it is crucial to acknowledge how the researcher's background and positionality influenced the research process. The researcher had previously worked with mental health and welfare family associations as an outside professional and instructor. This pre-existing role established a strong foundation of trust and credibility, which not only facilitated the snowball sampling recruitment but also fostered a safe, empathetic environment. Consequently, this relationship encouraged the participants to be highly open and share their vulnerable, detailed accounts without hesitation.

However, acknowledging this positionality, particularly the researcher's professional lens and inherent interest in the behaviors of parents and their impact on siblings, was essential to mitigate potential biases. To prevent these personal and professional values from overshadowing the data, the researcher rigorously documented all reflective thoughts and analytical judgments in theoretical memos, detailed written records of analytical thinking and decision-making, throughout the process. Furthermore, to ensure interpretation validity and maximum transparency, the findings were continuously peer-reviewed by co-researchers from diverse professional and familial backgrounds. Finally, regular supervision provided through the modified grounded theory approach study groups served as an objective mechanism to ensure that the final analytical model strictly prioritized and reflected the authentic perspectives of the participants.

### 2.3 Research participants

We solicited introductions from mental health and family welfare associations in three different prefectures in Japan. These prefectures encompass both urban and rural areas, ensuring that the participants represented diverse living environments and regional cultural contexts within the country, rather than being limited to a single local background. Because the siblings of individuals with schizophrenia often face social stigma and have lower active participation rates in family associations compared with their parents, they constitute a “hard-to-reach” population. Therefore, the use of snowball sampling was justified as an essential and effective strategy to access this hidden demographic through the trusted personal networks of initial contacts.

Although the initial recruitment criteria broadly targeted the adult siblings of individuals with schizophrenia, the sampling process coincidentally yielded a sample consisting entirely of married individuals with children. Although unintended, this homogeneity ultimately strengthened the study by providing a lens with which to focus on the specific dynamics of balancing caregiving for an affected sibling with the responsibilities of one's own family. Initially, eight individuals consented to participate. However, one individual, who provided written responses to prevent the spread of coronavirus disease 2019 (COVID-19), was excluded from the analysis because the contextual depth was insufficient. The remaining seven participants included one older brother, two older sisters, and four younger sisters of individuals with schizophrenia and had a mean age of 56.3 ± 6.2 years (range, 50–65 years) (Table 1).

**Table 1:** Characteristics of the participants and their siblings with schizophrenia.

Participants					Siblings with schizophrenia			
	Age	Relationship	Marital status	Children	Sex	Age	Duration of illness (years)	Current living situation
A	50s	Brother	Married	Yes	Male	50s	30	Living alone
B	50s	Older sister	Married	Yes	Female	40s	Unknown	Living alone
C	60s	Younger sister	Married	Yes	Female	60s	46	Living alone
D	60s	Younger sister	Married	Yes	Male	70s	50	Living alone
E	50s	Older sister	Married	Yes	Male	40s	11	Hospitalized
F	50s	Younger sister	Married	Yes	Female	50s	20	Living with husband
G	60s	Younger sister	Married	Yes	Female	60s	45	Hospitalized

### 2.4 Data collection and procedure

Data were collected between February 2019 and January 2020. Semi-structured interviews with the seven participants were conducted using an interview guide. During the interviews, the participants were encouraged to express themselves freely, and follow-up questions were asked based on the flow of the conversation. The interviews focused on several key themes, including the development of schizophrenia in the affected sibling, the factors that required them to assume responsibility for providing care, and their past interactions with both their parents and the affected sibling. Furthermore, the discussions explored the challenges they faced in providing support, as well as the individuals or circumstances that facilitated their caregiving efforts. The interviews lasted an average of 1 h and 22 min (range: 42 min to 2 h and 41 min). With the participants’ consent, the interviews were audio-recorded using a voice recorder and subsequently transcribed verbatim. Ultimately, these verbatim transcripts served as the data for the final analysis.

### 2.5 Data analysis

The data analysis was performed manually, without the use of qualitative data analysis software, utilizing the rigorous procedures of the modified grounded theory approach to ensure methodological reliability (Kinoshita, 2020). First, to define and limit the applicable scope of the constructed theory, we established the analytic focus as “married individuals who have a sibling with schizophrenia.” To operationalize the overarching research aim for this specific demographic, the analytic theme was set as “the process by which married individuals reconstruct their relationships with an affected sibling while maintaining their own lives as their parents age.”

The analysis began with the transcript that provided the most comprehensive relevant narratives. We utilized analysis worksheets to deeply interpret the interactive phases of the participants’ experiences. Through this deep interpretation, theme-relevant sections were extracted to generate initial concepts. In the modified grounded theory approach, a concept is the most fundamental component in the analysis, possessing concrete meaning strictly rooted in the data. Subsequently, we performed a continuous comparative analysis by deliberately seeking similar and contrasting examples both within the initial case and across other transcripts.

By rigorously examining the relationships among these generated concepts, interrelated concepts were systematically grouped into categories. A category is formed by identifying relationships between concepts through comparative analysis, interpreting them as hierarchies or orders, and expressing human behavior as “movements” composed of multiple concepts. Throughout this iterative process, theoretical memos were actively used to refine our interpretations.

This procedure culminated in the creation of a results diagram illustrating the structural process between the categories and concepts. Based on this diagram, a comprehensive storyline was crafted. The storyline is a concise written statement of the analytical findings, which represent the conclusions regarding the analysis theme, constructed using the categories and major concepts to explain the dynamic process of the phenomenon. Finally, theoretical saturation was determined to be reached when continuous comparison with new data yielded no new concepts or dimensions and the constructed diagram and storyline could comprehensively explain all individual variations and behaviors within the defined scope of the analytic theme.

We used the Standards for Reporting Qualitative Research reporting guideline (O’Brien et al., 2014) to draft this manuscript, and the Standards for Reporting Qualitative Research reporting checklist (O’Brien et al., 2025) when editing.

### 2.6 Ethical considerations

The study protocol was approved by the Medical Ethics Committee of Kanazawa University (Approval No. 655–1). The participants were provided with written and oral explanations of the study's overview, methods, benefits and risks, withdrawal rights, potential psychological stress from the interviews, support measures, and the protection of personal information. Only those who provided written informed consent were included in the study. Interviews were conducted in private rooms to ensure participant privacy.

## 3 Results

**Fig. 1:**
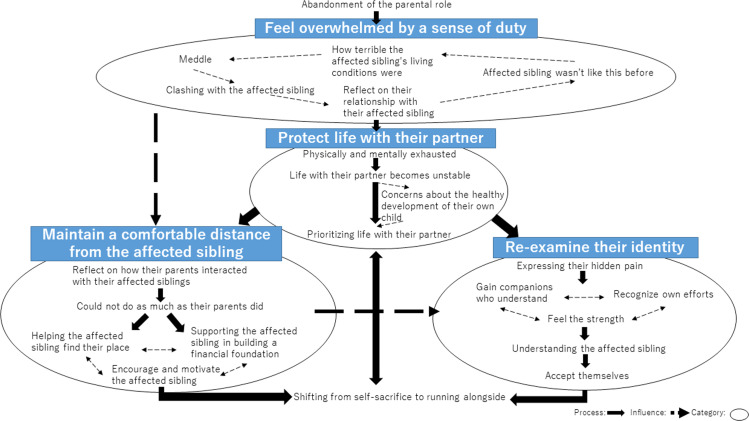
Process by which married individuals reconstruct their relationship with an affected sibling while maintaining their own lives as their parents age.

Twenty-two distinct concepts were derived from the participants’ narratives. In the following text, categories are denoted by **bold brackets []**, concepts by single quotation marks’, and the actual narratives by *italics*. Upon analyzing the interrelationships among these concepts, a central category was identified: **[Protect life with their partner]**. Furthermore, three other categories were generated: **[Feel overwhelmed by a sense of duty], [Maintain a comfortable distance from the affected sibling], and [Re-examine their identity]** (Fig. 1).

### 3.1 Storyline

The dynamic process by which married individuals reconstruct their relationship with a sibling who has schizophrenia is fundamentally driven by the need to balance their own family life with caregiving responsibilities. The process begins when the parents’ aging leads to the ‘abandonment of the parental role’. Consequently, the participants initially **[Feel overwhelmed by a sense of duty]**. As they attempt to intervene in their sibling’s life, they become ‘physically and mentally exhausted’ and their ‘life with their partner becomes unstable’. Facing this crisis of mutual destruction, they reach a critical turning point where they **[Protect life with their partner]** by consciously ‘prioritizing life with their partner’. Acknowledging their limitations, specifically that they ‘could not do as much as their parents did’, the unaffected siblings shift their support approach toward fostering independence, so that they **[Maintain a comfortable distance from the affected sibling]**. Concurrently, by ‘expressing their hidden pain’ in safe environments like family associations, they **[*Re*-examine their identity]**, which ultimately leads them to ‘accept themselves’. Through these interconnected processes, the participants effectively maintain their own lives while ‘shifting from self-sacrifice to running alongside’ the affected sibling.

### 3.2 [Feel overwhelmed by a sense of duty]

This category explains the initial phase of the overall process, which ultimately drives the participants toward the central category as their caregiving burdens threaten their own family life. The process is triggered by the ‘abandonment of the parental role’. Confronted with this sudden shift, married individuals unexpectedly assume full responsibility for their affected sibling. Consequently, the parents' consideration for their affected children is withdrawn.

*Parents often say they don’t want to bother their children, so they just live their lives telling them not to worry. However, when they can no longer manage it, they end up leaving everything to their children. … That responsibility just gets completely thrown onto us as it is*. (C)

Confronted with the reality of the situation, the married individuals are shocked to discover ‘how terrible the affected sibling’s living conditions were’. Driven by a sudden sense of responsibility, they try to ‘meddle’, which inevitably results in ‘clashing with the affected sibling’. Through these continuous conflicts, they take time to ‘reflect on their relationship with their affected sibling’ and are deeply distressed by the realization that their ‘affected sibling wasn’t like this before’.

*I’ve always thought that my younger sister is missing out on life. When I try to get her support, from her perspective, it is just her older sister who acts on her own. … Figuring out how to handle that part is honestly incredibly difficult.* (B)

### 3.3 [Protect life with their partner]

This category functions as the central category of the process, representing the critical turning point from self-sacrifice to self-preservation. Driven by the continuous conflicts and caregiving burdens experienced in the previous phase, the participants become ‘physically and mentally exhausted’.

*Actually, even now I’m not feeling well. I’m taking medication. I was told that I have depression. Back then, there were a lot of things going on, and I could not just cut back on work immediately… I was in a state where I had no strength left in me.* (E)

Consequently, their ‘life with their partner becomes unstable’, which subsequently raises ‘concerns about the healthy development of their own child’. Recognizing the destructive limits of self-sacrifice, they resolve this crisis by making the definitive choice of ‘prioritizing life with their partner’.

*My affected sibling was always prioritized. Everything was about them… My husband would say, “Shouldn’t your own children come first?” Yet I would rush over to my affected sibling. I realized that I did not want that life anymore. I made my child suffer because of it*. (B)

### 3.4 [Maintain a comfortable distance from the affected sibling]

This category describes how the married individuals establish a new, sustainable boundary, triggered by the central category’s definitive choice to protect their own families. They ‘reflect on how their parents interacted with the affected sibling’ and accept the reality that they ‘could not do as much as their parents did’. Based on this acknowledgment of their limitations, they reconstruct their approach by ‘helping the affected sibling find their place’ and ‘supporting the affected sibling in building a financial foundation’.

*Even if my wife understood and lived with my brother who has schizophrenia, I don’t think we could have had peaceful days. … If my own daughter did the same thing (developed the same illness and became a shut-in), I would feel responsible… But regarding my brother with schizophrenia, my position is far more complicated*. (A)

Through this balanced involvement, they ‘encourage and motivate the affected sibling’ without over-interfering, aiming to prevent mutual destruction.

*When you praise children, they grow. That is what I tell my older brother, too. Because he has that illness, he is pessimistic and feels pathetic. I tell him, “You don’t have to be so down about it.” … This is about helping him build confidence*. (D)

### 3.5 [*Re*-examine their identity]

This category illustrates the internal transformation facilitated by peer support, which occurs concurrently with the behavioral changes initiated by the central category. While adjusting their distance from their sibling, the married individuals begin ‘expressing their hidden pain’ in family associations. Through this self-disclosure, they ‘gain companions who understand’ them.

*I was at the venue for a family association seminar… I mustered courage and asked, “Would you mind telling me your story?” … When I listen to others’ stories, I get energy; we encourage each other, and it becomes a source of motivation for me as well*. (E)

This peer validation allows them to objectively ‘recognize own efforts’ and ‘feel the strength’. By ‘understanding the affected sibling’ from a more objective perspective, they finally overcome their internal struggles and ‘accept themselves’.

*My mother-in-law doesn’t understand anything about schizophrenia. However, she tells me that it’s inevitable for me to go and take care of my sister, and she recognizes that I’m working hard for her. She validates my efforts and has even supported me with the housework. Through this, I’ve also come to feel that I’m doing a good job myself.* (G)

*I don’t know who it will happen to or when, and the cause isn’t really known yet, so I sometimes think, maybe this could have happened to me instead. I suppose it’s not so much about understanding schizophrenia as it is about understanding my older sister who has schizophrenia.* (F)

## 4 Discussion

Previous research on family caregiving for schizophrenia has predominantly focused on the burden placed on parents, leaving the experiences of adult siblings largely unexplored. Although the limited literature on siblings has primarily emphasized caregiver burden and psychological distress, the primary novelty of the present study lies in its conceptualization of the dynamic process through which married individuals reconstruct their relationships with an affected sibling while maintaining their own lives as their parents age. Specifically, instead of merely assuming the parental role “self-sacrifice”, the unaffected siblings establish their own boundaries and transition into a new, sustainable relationship of “running alongside” the affected sibling. The following discussion explores the significance of this transition, particularly focusing on how this newly identified relationship of “running alongside” differs from traditional family caregiving models.

### 4.1 The stage where unaffected siblings [Feel overwhelmed by a sense of duty] and the turning point where they [Protect life with their partner]

When an unaffected sibling of an individual with schizophrenia becomes a primary caregiver, factors such as unilateral role assignment, inadequate illness comprehension, and pre-illness memories necessitate shifts in the sibling relationship. This change creates conflict and imposes psychological pressure on the married individual, often driven by the traditional societal expectation of “parental replacement,” in which siblings are assumed to simply inherit the self-sacrificing caregiving model of their parents (Kitzmüller et al., 2023; Yang et al., 2017). Graves et al. (2020) noted that unaffected siblings face conflict between caregiving demands and personal needs, termed “role engulfment” in Alzheimer’s caregiver studies, leading to reduced self-esteem and depression (Skaff and Pearlin, 1992). These siblings often lack information because their parents withheld details of the illness (Jayasinghe et al., 2023), contributing to this conflict by preventing an accurate understanding of the affected sibling’s schizophrenia. Plessis et al. (2020) found that 61.2% of siblings were unaware of their affected sibling’s specific illness, while Kitzmüller et al. (2023) noted that siblings may not understand the symptoms. Many grasp the conditions of their affected sibling only upon becoming primary caregivers. Pre-illness memories intensify this conflict, with more severe perceived symptoms leading to greater conflicts (Plessis et al., 2020).

Sibling interactions change through the need to [protect life with their partner], a boundary-setting process preventing mutual destruction while enabling long-term involvement. Dodge and Smith (2019) showed that balancing relationships with affected siblings and one’s own family determines the involvement level of the married individuals. This boundary setting is a conscious rejection of the traditional enmeshed caregiving role, facilitating the transition from “self-sacrifice” to “running alongside” the affected sibling.

### 4.2 [Maintain a comfortable distance from the affected sibling]: role renegotiation and new forms of connection

The recognition that married individuals cannot fulfill the same roles as their parents reflects a realistic understanding that assuming total parental responsibility is neither feasible nor necessary. Because these siblings transition into adulthood and form their own families, this realization involves a critical process of “role renegotiation” to balance competing familial demands and prevent caregiver burnout (Carter and McGoldrick, 1998; Cicirelli, 1995). Furthermore, viewed through the lens of family systems theory, this dynamic requires conscious “boundary setting” to maintain clear, healthy delineations between the family of origin and the new family (Minuchin, 1974; Rolland, 1999).

The newly identified concept of “running alongside” represents a sustainable middle ground. It clearly differentiates itself from the extreme enmeshment (over-involvement) seen in traditional self-sacrificing caregiving models, while also avoiding the opposite extreme of disengagement, laissez-faire, or abandonment of the affected sibling. This awareness constitutes an initial step in preventing burnout and facilitating sustainable support. Furthermore, studies indicate that the perception of having access to social support helps siblings of individuals with schizophrenia mitigate their pressure and enhance their sense of wellbeing (Avcıoğlu et al., 2019). Thus, utilizing social resources while maintaining a balanced distance, neither becoming overly involved nor disengaging, is emblematic of the “running alongside” approach. In conclusion, transitioning to a relationship where they [maintain a comfortable distance from the affected sibling] serves as a strategy to manage their parents’ aging. Through this approach, married individuals can safeguard their lives while sustaining a long-term, healthy relationship with the affected sibling.

### 4.3 [*Re-*examine their identity]: sublimation of experience and reconstruction of the self

According to Erikson (1968), adolescence and early adulthood constitute a critical phase for identity establishment in general human development. Because the onset of schizophrenia typically occurs during this period (World Health Organization, 2025), it significantly affects the identity formation of both the affected individuals and their unaffected siblings. Several factors help married individuals overcome the challenges of having a sibling with schizophrenia and renew their self-awareness. Support through interactions with others is crucial. Peer support is an essential resource that helps siblings perceive their experiences as normal and reduces feelings of social isolation (Sidhu et al., 2006). Evidence has shown that increased peer interaction correlates with reduced caregiver pressure, depression, anxiety, and isolation (Dillinger and Kersun, 2020; Feriante et al., 2022). Forming friendships with those who understand them enables objective reflection on personal struggles, while acknowledgment from others can alleviate self-sacrificial pain and help married individuals value their efforts.

Acceptance of the illness was identified as another significant factor. Acknowledging that schizophrenia is not anyone’s fault and embracing both the illness and its inevitable consequences as uncontrollable are vital for the psychological wellbeing of the siblings of affected individuals (Friedrich et al., 2008; McCann et al., 2021). Similarly, the present study found that appropriately recognizing the affected sibling’s condition as an illness facilitated understanding in the married individuals and alleviated their perceived pressure.

Through these processes, the married individuals enhanced their self-esteem and established new identities. They reinterpreted their involvement with their affected sibling not as traditional “self-sacrifice” but as “running alongside” them, and achieved self-acceptance by seeing the affected sibling not as a “problem” but as part of their lives. By redefining their roles, they successfully reconstructed an identity distinct from the traditional “primary caregiver” narrative. These processes illustrate the resilience and growth of siblings (Ran et al., 2023) and relate to “post-traumatic growth,” which denotes positive psychological changes following adversity (Bitar et al., 2024). “Self-compassion,” defined as showing kindness to oneself by accepting one’s imperfections (Germer and Neff, 2013; Neff, 2023), enhances post-traumatic growth (Adonis et al., 2025). In the present study, it facilitated positive psychological changes in the participants.

### 4.4 Implications for clinical practice

The findings of this study offer insights into supporting married individuals who have a sibling with schizophrenia. As their parents age, the siblings’ evolving roles must be evaluated by healthcare professionals in order to equip them with the information and psychological support they need. The guilt experienced by the married individuals when choosing to [Protect life with their partner] should be mitigated, and their decisions should be respected. Social resources should be promoted to help them [Maintain a comfortable distance from the affected sibling]. Importantly, healthcare professionals must avoid projecting traditional parental caregiving expectations onto the married individuals who have a sibling with schizophrenia. Rather than positioning the married individuals as the sole primary caregivers, validating their boundary-setting and reinforcing their role as supportive companions helps the affected sibling achieve independence in society. Peer support groups and individual counseling opportunities are essential for them to [*Re*-examine their identity]. By sharing experiences and receiving empathy, caregiving siblings can reduce isolation, accept their emotions, and develop new self-awareness, thereby contributing to their long-term wellbeing.

### 4.5 Limitations

This study has some limitations. First, due to constraints on the number of participants, the participants were recruited without controlling for diversity in life stage or family structure, the progression of the affected siblings’ illnesses, or the participants’ gender. Second, the influence of gender norms and cultural backgrounds must be considered. Six of the seven participants in this study were women. In the Japanese cultural context, there remains a deep-rooted gender role expectation that women should act as the primary caregivers for aging parents and family members with disabilities. Consequently, the degree of “self-sacrifice” and the experience where they [Feel overwhelmed by a sense of duty] may strongly reflect these gender-specific social pressures. Third, there is a potential recruitment bias. Because the participants were recruited through mental health and family welfare associations, they may represent a highly resilient group who are already connected to support networks and capable of objectifying their situations. Individuals who are socially isolated and do not belong to such groups may face more severe self-sacrificing situations, and thus the identified process might not apply to them.

## 5 Conclusion

As their parents age, the married individuals who have a sibling with schizophrenia undergo a dynamic process of relationship reconstruction. Triggered by the critical need to **Protect life with their partner**, they transition from a state where they **Feel overwhelmed by a sense of duty** to a sustainable relationship of “running alongside” their affected sibling. Acknowledging that they cannot fully assume the parental role, they **Maintain a comfortable distance from the affected sibling** and ***Re-*examine their identity** through peer support. Ultimately, they are able to maintain continuous support without sacrificing their own well-being. Therefore, nursing professionals should facilitate this transition by supporting married individuals who have a sibling with schizophrenia in reconstructing their identities and balancing caregiving with their own lives, in addition to providing information, psychological support, social resources, and opportunities for peer interactions.

Future research should explore the effectiveness of specific nursing interventions designed to facilitate this transition toward “running alongside” the affected sibling. Additionally, further studies are needed to examine the caregiving processes of siblings across different cultural contexts and more diverse demographic backgrounds, particularly including more male siblings and those not affiliated with support groups.

## Unblinded ethical approval

This study was approved by the Medical Ethics Committee of Kanazawa University (Approval No. 655–1) and followed an approved protocol. The participants were provided with written and verbal explanations of the study's overview, methods, benefits and risks, withdrawal rights, potential psychological stress from interviews, support measures, and personal information protection. Only those who provided written informed consent were included in this study. Interviews were conducted in private rooms to ensure participant privacy.

## Funding

This research received partial support from JSPS KAKENHI Grant Number JP24K13774.

## CRediT authorship contribution statement

**Megumi Kawaguchi:** Writing – original draft, Visualization, Validation, Resources, Project administration, Methodology, Investigation, Funding acquisition, Formal analysis, Data curation, Conceptualization. **Miho Katayama:** Writing – review & editing, Validation. **Akiyo Nakamoto:** Writing – review & editing, Validation. **Hiromi Morioka:** Validation, Project administration. **Midori Kawamura:** Validation.

## Declaration of competing interest

The authors declare that they have no known competing financial interests or personal relationships that could have appeared to influence the work reported in this paper.

## Data Availability

The datasets used and/or analysed during the current study are available from the corresponding author on reasonable request.

## References

[bib0001] Adonis M., Loucaides M., Sullman M.J.M., Lajunen T. (2025). The protective role of self compassion in trauma recovery and its moderating impact on post traumatic symptoms and post traumatic growth. Sci. Rep..

[bib0002] Amaresha A.C., Venkatasubramanian G., Muralidhar D. (2019). Perspectives about illness, attitudes, and caregiving experiences among siblings of persons with schizophrenia: a qualitative analysis. Indian J. Psychol. Med..

[bib0003] Avcıoğlu M.M., Karanci A.N., Soygur H. (2019). What is related to the well-being of the siblings of patients with schizophrenia: an evaluation within the Lazarus and Folkman’s Transactional Stress and Coping Model. Int. J. Soc. Psychiatry..

[bib0004] Bitar Z., Fekih-Romdhane F., Mahfoud D., Fawaz M., Hallit S., Obeid S. (2024). The mediating effect of post-traumatic growth on the relationship between personality traits and resilience among a sample of Lebanese adults. PloS One.

[bib0005] Carter B., McGoldrick M. (1998). The Expanded Family Life cycle: individual, family, and Social Perspectives.

[bib0006] Casale E.G., Burke M.M., Urbano R.C., Arnold C.K., Hodapp R.M. (2021). Getting from here to there: future planning as reported by adult siblings of individuals with disabilities. J. Intellect. Disabil. Res..

[bib0007] Chiu C.-Y. (2022). Bamboo sibs: experiences of Taiwanese non-disabled siblings of adults with intellectual and developmental disabilities across caregiver lifestages. J. Dev. Phys. Disabil..

[bib0008] Cicirelli V.G. (1995).

[bib0009] Dillinger R.L., Kersun J.M. (2020). Caring for caregivers: understanding and meeting their needs in coping with first episode psychosis. Early Interv. Psychiatry..

[bib0010] Dodge C.E., Smith A.P. (2019). Caregiving as role transition: siblings’ experiences and expectations when caring for a brother or sister with schizophrenia. Can. J. Community Ment. Health..

[bib0011] Erikson E.H. (1968).

[bib0012] Feriante J., Shayani A., Lauer E., Pressman A., Rubin E. (2022). Sibling support program: a novel peer support intervention for parents, caregivers and siblings of youth experiencing mental illness. Healthcare (Basel).

[bib0013] Forcheron V., Sacareau E., Bourgeois J., Pouchon A., Polosan M., Gaboreau Y., Dondé C. (2023). Experience, impact and needs of informal parental caregivers around the communication of a diagnosis of schizophrenia. Int. J. Soc. Psychiatry..

[bib0014] Friedrich R.M., Lively S., Rubenstein L.M. (2008). Siblings' coping strategies and mental health services: a national study of siblings of persons with schizophrenia. Psychiatr. Serv..

[bib0015] Gerace L.M., Camilleri D., Ayres L. (1993). Sibling perspectives on schizophrenia and the family. Schizophr. Bull..

[bib0016] Germer C.K., Neff K.D. (2013). Self-compassion in clinical practice. J. Clin. Psychol..

[bib0017] Graves J.M., Marsack-Topolewski C.N., Shapiro J. (2020). Emerging adult siblings of individuals with schizophrenia: experiences with family crisis. J. Fam. Issues..

[bib0018] Harrison G., Hopper K., Craig T., Laska E., Siegel C., Wanderling J., Dube K.C., Ganev K., Giel R., an der Heiden W., Holmberg S.K., Janca A., Lee P.W., León C.A., Malhotra S., Marsella A.J., Nakane Y., Sartorius N., Shen Y., Skoda C., Thara R., Tsirkin S.J., Varma V.K., Walsh D., Wiersma D. (2001). Recovery from psychotic illness: a 15- and 25-year international follow-up study. Br. J. Psychiatry..

[bib0019] Hsiao C.Y., Tsai Y.F. (2021). The experiences of adult siblings of people with schizophrenia: a systematic review and meta-synthesis of qualitative studies. Int. J. Ment. Health Nurs..

[bib0020] Jayasinghe A., Wrobel A., Filia K., Byrne L.K., Melvin G., Murrihy S., Moller C., Berk L., Berk M., Cotton S. (2023). Distress, burden, and wellbeing in siblings of people with mental illness: a mixed studies systematic review and meta-analysis. Psychol. Med..

[bib0021] Kim M.A., Yi J., Hwang S., Sung J., Lee S.Y., Kim H. (2025). Narratives from female siblings of adults with intellectual and developmental disabilities: a photovoice study on identity and growth experiences in South Korea. J. Appl. Res. Intellect. Disabil..

[bib0022] Kinoshita Y. (2020).

[bib0023] Kitzmüller G., Wiklund Gustin L., Kalhovde A.M. (2023). Filling the void: the role of adult siblings caring for a brother or sister with severe mental illness. Glob. Qual. Nurs. Res..

[bib0024] Lee H., Kim K., Kim H., Choi E.K. (2025). Experiences of siblings of individuals with developmental disabilities: a meta-synthesis of qualitative studies. Disabil. Health J..

[bib0025] Ma M., Shi Z., Chen Y., Ma X. (2023). Recovery journey of people with a lived experience of schizophrenia: a qualitative study of experiences. BMC Psychiatry.

[bib0026] Maan N., Kumar A., Nayar N., Kumar K., Sheoran C. (2024). Burden of care in caregivers of patients with schizophrenia in Greater Noida, U. P., India. J. Family Med. Prim. Care..

[bib0027] Manesh A.E., Dalvandi A., Zoladl M. (2023). The experience of stigma in family caregivers of people with schizophrenia spectrum disorders: a meta-synthesis study. Heliyon.

[bib0028] McCann E., Lee R., Brown M. (2021). The experiences of young adult siblings of individuals with psychosis: an interpretative phenomenological analysis. J. Psychiatr. Ment. Health Nurs..

[bib0029] Milevsky A., Singer O. (2022). Growing up alongside a sibling with a disability: a phenomenological examination of growth and deficiency in adulthood. Res. Dev. Disabil..

[bib0030] Milliken P.J. (2001). Disenfranchised mothers: caring for an adult child with schizophrenia. Health Care Women Int..

[bib0031] Minuchin S. (1974).

[bib0032] Neff K.D. (2023). Self-compassion: theory, method, research, and intervention. Annu. Rev. Psychol..

[bib0033] O’Brien B.C., Harris I.B., Beckman T.J., Reed D.A., Cook D.A. (2014). Standards for reporting qualitative research: a synthesis of recommendations. Acad. Med..

[bib0034] O’Brien B.C., Harris I.B., Beckman T.J., Reed D.A., Cook D.A., Harwood J., Albury C., de Beyer J., Schlüssel M., Collins G. (2025). The EQUATOR Network Reporting Guideline Platform.

[bib0035] Plessis L., Wilquin H., Pavani J.B., Bouteyre E. (2020). Explaining differences between sibling relationships in schizophrenia and nonclinical sibling relationships. Front. Psychiatry..

[bib0036] Ran M.S., Chui E.W.T., Shin S. (2023). Resilience of siblings of people with mental illness: a systematic review. Int. *J. Soc. Psychiatry*.

[bib0037] Rolland J.S. (1999). Parental illness and disability: a family systems framework. J. Fam. Ther..

[bib0038] Sharifi M., Younesi S.J., Foroughan M., Safi M.H., Khanjani M.S. (2023). The challenges of caring for an adult child with schizophrenia in the family: an analysis of the lived experiences of older parents. Inquiry.

[bib0039] Sidhu R., Passmore A., Baker D. (2006). The effectiveness of a peer support camp for siblings of children with cancer. Pediatr. Blood Cancer..

[bib0040] Skaff M.M., Pearlin L.I. (1992). Caregiving: role engulfment and the loss of self. Gerontologist.

[bib0041] Steel J. (2019). The sibling’s role in schizophrenia care: a neglected reality. J. Fam. Nurs..

[bib0042] World Health Organization (2025). https://www.who.int/news-room/fact-sheets/detail/schizophrenia.

[bib0043] Yang C.I., Hsieh M.Y., Lee L.H., Chen S.L. (2017). Experiences of caring for a sibling with schizophrenia in a Chinese context: a neglected issue. Int. J. Ment. Health Nurs..

